# Staphylococcus aureus Releases Proinflammatory Membrane Vesicles To Resist Antimicrobial Fatty Acids

**DOI:** 10.1128/mSphere.00804-20

**Published:** 2020-09-30

**Authors:** Arnaud Kengmo Tchoupa, Andreas Peschel

**Affiliations:** a Department of Infection Biology, Interfaculty Institute for Microbiology and Infection Medicine Tübingen (IMIT), University of Tübingen, Tübingen, Germany; b Cluster of Excellence “Controlling Microbes to Fight Infections,” University of Tübingen, Tübingen, Germany; University of Nebraska Medical Center

**Keywords:** *Staphylococcus aureus*, Toll-like receptors, antimicrobial fatty acids, lipoproteins, membrane vesicles

## Abstract

Staphylococcus aureus is a major pathogen, which colonizes one in three otherwise healthy humans. This significant spread of S. aureus is largely due to its ability to circumvent innate immune responses, including antimicrobial fatty acids (AFAs) on the skin and in nasal secretions. In response to AFAs, S. aureus swiftly induces resistance mechanisms, which have yet to be completely elucidated. Here, we identify membrane vesicle (MV) release as a resistance strategy used by S. aureus to sequester host-specific AFAs. MVs protect S. aureus against a wide array of AFAs. Strikingly, beside MV production, S. aureus modulates MV composition upon exposure to AFAs. MVs purified from bacteria grown in the presence of linoleic acid display a distinct protein content and are enriched in lipoproteins, which strongly activate Toll-like receptor 2 (TLR2). Cumulatively, our findings reveal the protective capacities of MVs against AFAs, which are counteracted by an increased TLR2-mediated innate immune response.

**IMPORTANCE** The nares of one in three humans are colonized by Staphylococcus aureus. In these environments, and arguably on all mucosal surfaces, bacteria encounter fatty acids with antimicrobial properties. Our study uncovers that S. aureus releases membrane vesicles (MVs) that act as decoys to protect the bacterium against antimicrobial fatty acids (AFAs). The AFA-neutralizing effects of MVs were neither strain specific nor restricted to one particular AFA. Hence, MVs may represent “public goods” playing an overlooked role in shaping bacterial communities in AFA-rich environments such as the skin and nose. Intriguingly, in addition to MV biogenesis, S. aureus modulates MV composition in response to exposure to AFAs, including an increased release of lipoproteins. These MVs strongly stimulate the innate immunity via Toll-like receptor 2 (TLR2). TLR2-mediated inflammation, which helps to fight infections, may exacerbate inflammatory disorders like atopic dermatitis. Our study highlights intricate immune responses preventing infections from colonizing bacteria.

## Abstract (precis)

The nares of one in three humans are colonized by Staphylococcus aureus. In these environments, and arguably on all mucosal surfaces, bacteria encounter fatty acids with antimicrobial properties. Our study uncovers that S. aureus releases membrane vesicles (MVs) that act as decoys to protect the bacterium against antimicrobial fatty acids (AFAs). The AFA-neutralizing effects of MVs were neither strain specific nor restricted to one particular AFA. Hence, MVs may represent “public goods” playing an overlooked role in shaping bacterial communities in AFA-rich environments such as the skin and nose. Intriguingly, in addition to MV biogenesis, S. aureus modulates MV composition in response to exposure to AFAs, including an increased release of lipoproteins. These MVs strongly stimulate the innate immunity via Toll-like receptor 2 (TLR2). TLR2-mediated inflammation, which helps to fight infections, may exacerbate inflammatory disorders like atopic dermatitis. Our study highlights intricate immune responses preventing infections from colonizing bacteria.

## INTRODUCTION

Staphylococcus aureus is a Gram-positive bacterium and the causative agent of numerous infections ranging from mild skin and soft tissue infections to invasive infections, such as bacteremia, endocarditis, and pneumonia (1). The morbidity, mortality, and health costs of these infections are exacerbated by the high prevalence of multidrug-resistant strains (2). In contrast, S. aureus colonizes asymptomatically the nares of ∼30% of the human population (3). For this bacterium, the skin carriage differs sharply between healthy individuals (5 to 20%) and patients with skin disorders such as atopic dermatitis (80 to 100%) (4). Both skin and nasal environments are rich in long-chain unsaturated fatty acids with antimicrobial properties (5, 6). These antimicrobial fatty acids (AFAs) also contribute to an important defense mechanism against pathogens by maintaining the low pH of the skin, whose alkalization correlates with microbial dysbiosis, increased colonization with S. aureus, and atopic dermatitis (7, 8).

In mice, topical application, intraperitoneal injection, and AFA-rich diet lead to decreased bacterial load and increased survival upon S. aureus infections (9, 10). AFAs do not inhibit only S. aureus and numerous Gram-positive species but also Gram-negative bacteria (11, 12). However, the role of AFAs in the innate immune response to bacterial infections goes beyond direct toxicity. Indeed, AFAs also possess immune-modulatory properties. For instance, upon incubation with sebum AFAs, human sebocytes considerably enhance their expression and secretion of beta-defensin 2, one of the predominant antimicrobial peptides found in the skin (13). Neutrophil release of the antimicrobial peptide LL-37 and alpha-defensins is also stimulated by AFAs (14). Furthermore, AFA incorporation into S. aureus lipoproteins potentiates TLR2 (Toll-like receptor 2)-dependent innate immune activation (15).

The S. aureus membrane is the primary target of AFAs, and their effects include increased fluidity, compromised integrity, and depolarization (16, 17). Recently, arachidonic acid, a polyunsaturated AFA, was shown to kill S. aureus via lipid peroxidation (18). In response to the pleiotropy of AFA toxicity, S. aureus has developed numerous resistance strategies. For instance, under iron-limiting conditions, the surface protein IsdA increases S. aureus cellular hydrophilicity, precluding the bacterial binding to hydrophobic AFAs (9). Owing to similar properties, wall teichoic acids shield S. aureus against AFAs (19). However, efflux pumps FarE (20) and Tet38 (21) prevent the cellular accumulation of AFAs, which are still able to bind to S. aureus. Additionally, this bacterium possesses a functional oleate hydratase, which hydrates and thereby detoxifies AFAs containing *cis*-9 double bonds (22).

Strikingly, S. aureus grown for a few hours in the presence of subinhibitory amounts of AFAs survives subsequent exposures to otherwise bactericidal AFA concentrations (20, 23), suggesting that the bacteria activate an AFA stress response program. High-throughput transcriptomic and proteomic studies on S. aureus primed with AFAs revealed more than 100 differentially expressed genes (17, 23–25) but could not identify an inducible AFA resistance mechanism common to all S. aureus strains and efficient against various AFAs. However, S. aureus primed with several AFAs secretes distinct proteins, including triacylglycerol lipase 2 and several proteases (23, 26). Intriguingly, the contribution of secreted factors to S. aureus resistance has not been thoroughly investigated. In addition to oleate hydratase, we sought to identify further factors released by S. aureus to neutralize AFAs.

Here, using a clickable AFA analogue, we show that S. aureus-conditioned medium sequesters AFAs and prevents their binding to the bacteria. Furthermore, we characterize the AFA-binding capacity of S. aureus membrane vesicles (MVs), which enable the bacteria to grow in the presence of otherwise toxic amounts of AFAs. In response to AFAs, S. aureus modulates its MV production and composition. MVs released in the presence of linoleic acid (LA) are enriched in lipoproteins and induce a potent TLR2 stimulation. Thus, the protective effects of MVs against AFAs are counteracted by a stronger innate immune response.

## RESULTS

### S. aureus release decoys that reduce AFA bacterial binding.

**FIG 1:**
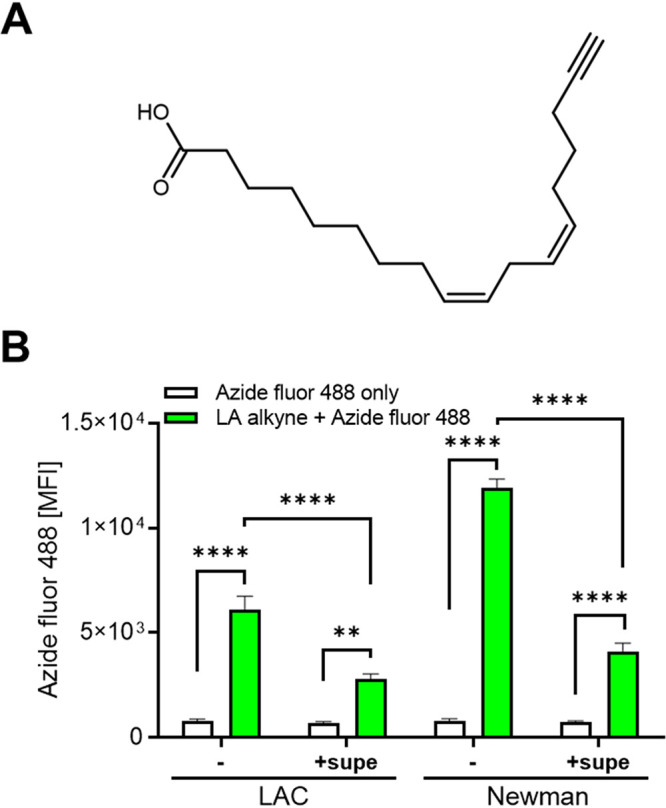
Culture supernatants impede S. aureus targeting by LA alkyne. (A) Chemical structure of alkyne functionalized linoleic acid (LA alkyne; 9*Z*,12*Z*-octadecadien-17-ynoic acid). (B) S. aureus bacteria grown for 6 h in TSB were incubated at 37°C for 20 min with or without LA alkyne prior to labeling with azide fluor 488 and flow cytometry analyses. Click chemistry was performed in the absence (-) or presence of culture supernatants (+supe). Data shown are bacterial mean fluorescence intensities (MFI) plus standard errors of the means (SEM) (error bars) (*n *= 3). Values that are significantly different by one-way ANOVA with Tukey’s test are indicated by asterisks as follows: **, *P* < 0.01; ****, *P* < 0.0001.

To gain new insights into S. aureus interaction with AFAs, we used a LA analogue, linoleic acid alkyne (Fig. 1A), and click chemistry with azide fluor 488 for AFA-binding studies. Importantly, LA retained its capacity to inhibit S. aureus growth upon addition of the alkyne group compared to LA (see Fig. S1A and B in the supplemental material). LA alkyne binding to S. aureus USA300 LAC and Newman strains, which are resistant and sensitive to methicillin, respectively, could be readily quantified by flow cytometry (Fig. 1B). Strikingly, bacteria stained with LA alkyne in the presence of S. aureus-conditioned culture supernatants exhibited markedly decreased signals compared to bacteria resuspended in fresh medium, suggesting that S. aureus releases a secreted factor to its culture supernatant that sequesters LA or interferes otherwise with LA binding. We extended our LA-binding studies to two other S. aureus strains: USA400 MW2 (community-acquired methicillin-resistant S. aureus [MRSA]) and SH1000 (methicillin-susceptible S. aureus [MSSA]). Clearly, a strong decrease in LA staining upon click chemistry in the presence of bacterial supernatants was noticed for both strains compared to fresh medium (Fig. S2). Taken together, our data suggest that S. aureus impedes binding by AFAs by a new strategy involving a secreted factor.

10.1128/mSphere.00804-20.1 FIG S1 Linoleic acid alkyne inhibits S. aureus growth. (A and B) S. aureus LAC (A) or Newman (B) was grown in the presence or absence of 100 μM LA or LA alkyne. Data, represented as area under the curve, are means ± SEM; *n *= 3; **, *P* < 0.01, ****, *P* < 0.0001, using one-way ANOVA with Dunnett’s test. Download FIG S1, TIF file, 0.2 MB. Copyright © 2020 Kengmo Tchoupa and Peschel. 2020 Kengmo Tchoupa and Peschel This content is distributed under the terms of the Creative Commons Attribution 4.0 International license.

10.1128/mSphere.00804-20.2 FIG S2 S. aureus*-*conditioned medium prevents bacterial binding by linoleic acid alkyne. S. aureus MW2 and SH1000 strains grown for 6 h in TSB were incubated at 37°C for 20 min with or without LA alkyne prior to labeling with azide fluor 488 and flow cytometry analyses. Click chemistry was performed in the absence (-) or presence (+) of culture supernatants. Data shown are bacterial mean fluorescence intensities (MFI) ± standard errors of the means (SEM); *n *= 3; ****, *P* < 0.0001 using one-way ANOVA with Tukey’s test. Download FIG S2, TIF file, 0.2 MB. Copyright © 2020 Kengmo Tchoupa and Peschel. 2020 Kengmo Tchoupa and Peschel This content is distributed under the terms of the Creative Commons Attribution 4.0 International license.

### MVs promote S. aureus growth in the presence of AFAs.

**FIG 2:**
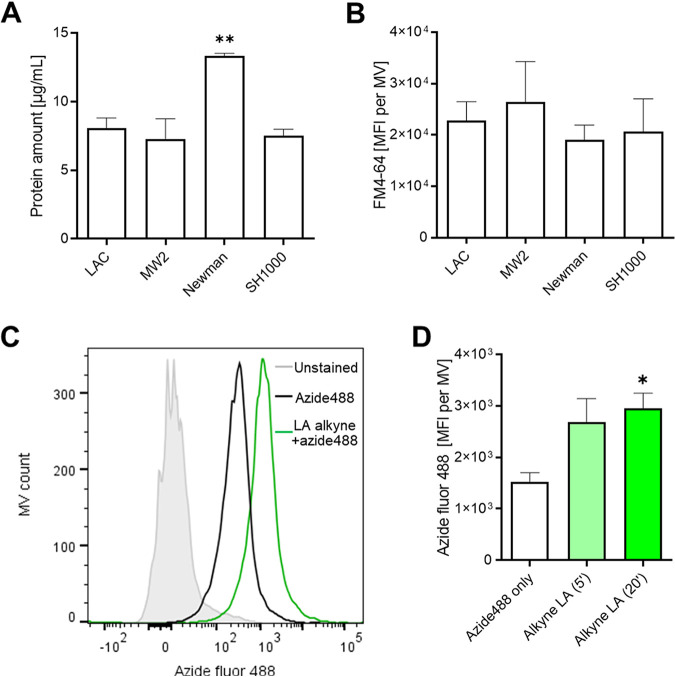
S. aureus releases MVs that can bind LA alkyne. (A) MV preparations from the indicated strains were analyzed for protein amounts per milliliter of bacterial cultures. (B) MVs were stained with FM4-64, and their mean fluorescence intensities (MFI) were analyzed by flow cytometry. (C) Representative flow cytometry histograms depicting a fluorescence shift upon staining with azide fluor 488 (azide488) for MVs pretreated with or without LA alkyne. Unstained MVs were used as controls. (D) MFI were obtained as described above for panel C for MVs treated with azide488 alone or in combination with LA alkyne for 5 or 20 min. Data shown as bar graphs are means plus SEM (*n *= 3). Statistical significance by one-way ANOVA with Dunnett’s test: *, *P* < 0.05; **, *P* < 0.01.

Recently, Andreoni and coworkers showed that the release of MVs helps S. aureus to survive exposure to daptomycin, a membrane-targeting antibiotic (27). Our finding that S. aureus releases a secreted factor that prevents bacterial accumulation of membrane targeting, labeled LA (Fig. 1) raised the question whether MVs may be responsible for sequestering AFAs in culture supernatants. First, we isolated MVs from MSSA and MRSA. S. aureus Newman MVs had the highest protein content (Fig. 2A), while all S. aureus MVs had similar lipid amounts, as measured with the lipophilic dye FM4-64 (Fig. 2B). As exemplified with the Newman strain, S. aureus MVs were highly hydrophobic and able to bind LA alkyne within a few minutes (Fig. 2C and D).

**FIG 3:**
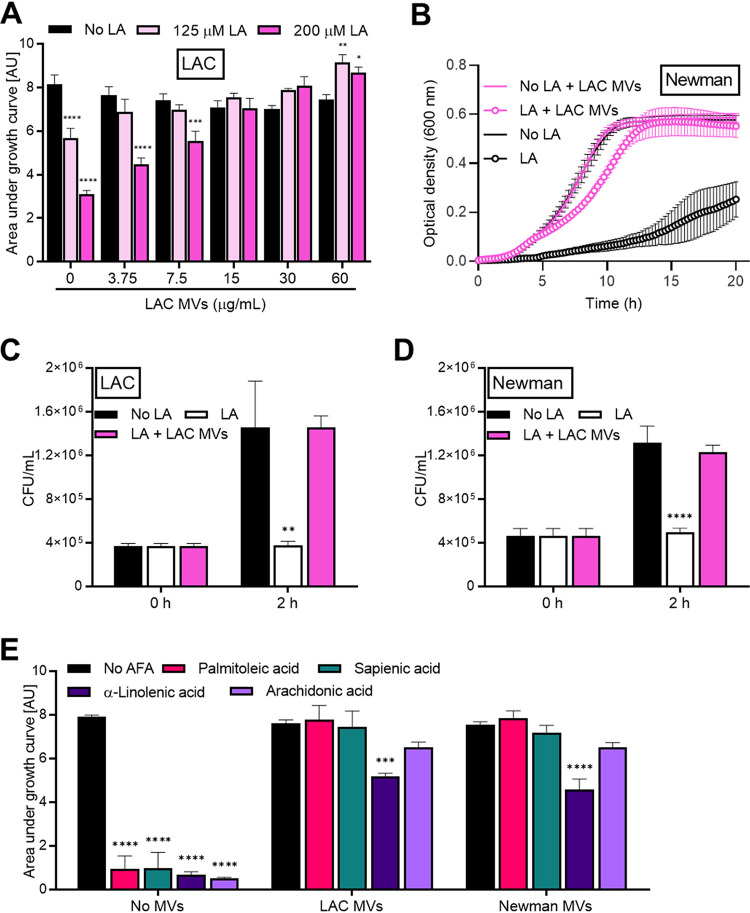
MVs confer a broad protection against AFAs. (A) Area under the curves of S. aureus LAC grown with or without linoleic acid (LA) in the presence of 0 to 60 μg/ml LAC MVs. AU, arbitrary units. (B) The growth of S. aureus Newman treated with 100 μM LA or left untreated was monitored with or without 30 μg/ml LAC MVs. (C and D) Viable bacteria upon 2-h growth with no LA or with LA or with LA plus 30 μg/ml LAC MVs were enumerated for LAC (C) and Newman (D) strains. (E) Area under the curves of LAC grown with or without 100 μM concentration of the indicated AFA in the presence or absence of LAC or Newman MVs. Means plus SEM are shown for at least three biological replicates. Statistical significance by two-way ANOVA with Tukey’s test for each time point (C and D) or growth condition (A and E): *, *P* < 0.05; **, *P* < 0.01; ***, *P* < 0.001; ****, *P* < 0.0001.

AFA-binding capacity of MVs prompted us to test whether MVs promote S. aureus growth in the presence of toxic amounts of AFAs. The growth of S. aureus strain LAC was inhibited by 125 or 200 μM LA, but growth was not impeded when bacteria were supplemented with MVs from the same strain (Fig. 3A). Importantly, S. aureus LAC MVs were also able to support S. aureus Newman growth in the presence of inhibitory amounts (100 μM) of LA, as revealed by optical density monitoring (Fig. 3B). In addition to optical density, CFU counts showed that LA toxicity was alleviated by LAC MVs for LAC (Fig. 3C) and Newman (Fig. 3D) strains. Likewise, Newman MVs abrogated the LA-induced growth inhibition of both LAC and Newman strains (Fig. S3A and B).

10.1128/mSphere.00804-20.3 FIG S3 MVs protect S. aureus against linoleic acid. (A and B) The growth of S. aureus LAC (A) or Newman (B) treated with or without LA was monitored in the presence or absence of 30 μg/ml Newman MVs. Shown are means ± SEM; *n *= 3. (C) *S aureus* LAC, treated with or without 200 μM LA, was grown in the absence (No MVs) or presence of LAC MVs isolated from bacteria grown in basic medium (BM MVs), lysogeny broth (LB MVs), Mueller-Hinton broth (MHB MVs), or TSB (TSB MVs). Data, represented as area under the curve, are means ± SEM; *n *= 3; ****, *P* < 0.0001 using two-way ANOVA with Sidak’s test for each growth condition. Download FIG S3, TIF file, 1.0 MB. Copyright © 2020 Kengmo Tchoupa and Peschel. 2020 Kengmo Tchoupa and Peschel This content is distributed under the terms of the Creative Commons Attribution 4.0 International license.

To make sure that the protective effect of MV preparations against LA was not due to some residual components of the complex media used to grow bacteria prior to MV isolation, LAC MVs were purified from bacteria grown in four different broths (basic medium, lysogeny broth, Mueller-Hinton broth [MHB], or tryptic soy broth [TSB]). These four types of LAC MVs equally helped the LAC strain grow in the presence of otherwise inhibitory amounts of LA (Fig. S3C).

To elucidate whether the AFA-neutralizing capacity of MVs is specific for LA, we investigated whether MVs could enable S. aureus growth in the presence of other AFAs in addition to LA. Accordingly, the LAC strain was grown with 100 μM palmitoleic, sapienic, α-linolenic, or arachidonic acid. These AFAs were all able to inhibit bacterial growth, which resumed in the presence of LAC or Newman MVs (Fig. 3E). However, MVs did not completely shield bacteria against α-linolenic acid (Fig. 3E and Fig. S4). Collectively, our data strongly suggest MVs as a resistance mechanism against a broad range of different AFAs.

10.1128/mSphere.00804-20.4 FIG S4 MVs enable S. aureus growth in the presence of various AFAs. *S aureus* LAC was grown without any AFA or with 100 μM palmitoleic acid (PA) (A and B), sapienic acid (SA) (C and D), α-linolenic acid (ALA) (E and F), or arachidonic acid (AA) (G and H). Some samples were supplemented with either LAC MVs (A, C, E, and G) or Newman MVs (B, D, F, and H). Shown are means ± SEM; *n *= 3. Download FIG S4, TIF file, 1.8 MB. Copyright © 2020 Kengmo Tchoupa and Peschel. 2020 Kengmo Tchoupa and Peschel This content is distributed under the terms of the Creative Commons Attribution 4.0 International license.

### Linoleic acid boosts S. aureus release of MVs with strong TLR2-stimulating capacities.

**FIG 4:**
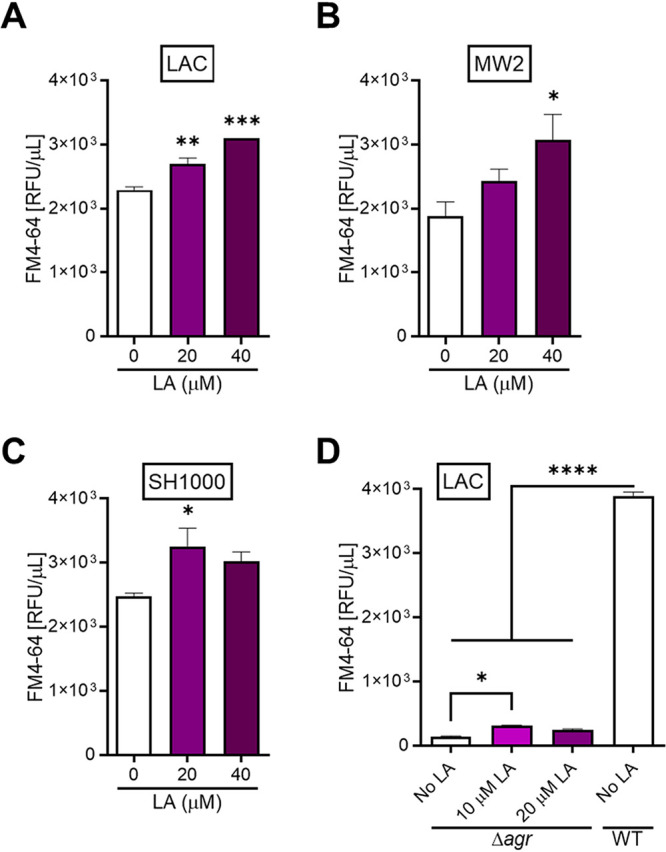
S. aureus boosts MV release in response to LA. (A to C) The same volume of MV preparations from similarly grown bacteria in TSB supplemented with 0, 20, or 40 μM LA was treated with FM4-64, and the stained MVs were quantified by fluorometry for strains LAC (A), MW2 (B), and Newman (C). (D) MV preparations from wild-type (WT) LAC and its isogenic *agr* mutant (Δ*agr*) grown with or without LA were analyzed as described above for panel A. Shown are means plus SEM for three biological replicates. Statistical significance by one-way ANOVA with Dunnett’s test (A to C) or Tukey’s test (D): *, *P* < 0.05; **, *P* < 0.01; ***, *P* < 0.001; ****, *P* < 0.0001.

S. aureus resistance to AFAs is known to be induced by subinhibitory amounts of AFAs (20, 23), which is concomitant with an altered secretome (23, 26). Our discovery that MVs protect S. aureus against AFA toxicity prompted us to analyze bacterial MV release in the presence of LA. As the presence of membrane lipids is a hallmark for MVs, MVs from S. aureus grown in the presence of 0, 20, or 40 μM LA were stained with the lipophilic dye FM4-64, and the amount of lipids in MV preparations (surrogate for MV amount) was quantified with a plate reader. S. aureus strains USA300 LAC (Fig. 4A), USA400 MW2 (Fig. 4B), and SH1000 (Fig. 4C) responded to LA exposure with a significantly increased release (20 to 60%) of AFA-neutralizing MVs. Thus, S. aureus appears to produce MVs as inducible decoys for the sequestration of harmful AFAs.

Recently, we demonstrated that the release of S. aureus MVs was driven by surfactant-like small peptides, phenol-soluble modulins (PSMs), which are controlled by the global virulence regulator Agr (28). Importantly, Agr-deficient mutants, which are defective in MV biogenesis, are also more susceptible to AFAs (24). LAC Δ*agr* significantly augmented its MV release (70 to 110%) in the presence of LA (Fig. 4D). However, irrespective of LA treatment, Δ*agr* MVs were residual compared to those of the wild type (Fig. 4D). Although a detailed mechanism of AFA-triggered increase in MV release is lacking, it is apparent that AFAs only enhance preexisting bacterial capacity to vesiculate.

**FIG 5:**
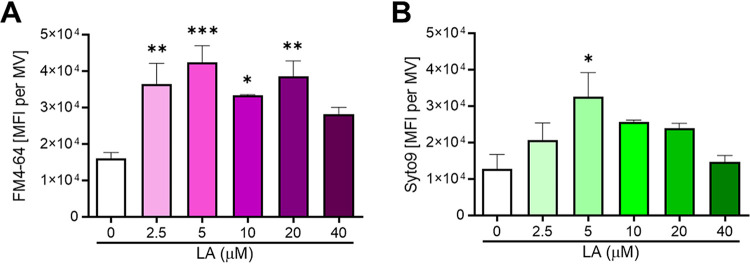
S. aureus modulates MV composition upon LA exposure. (A and B) MVs from S. aureus USA300 LAC grown in the presence of 0 to 40 μM LA were stained with FM4-64 (A) or SYTO 9 (B), and analyzed by flow cytometry to determine MFI per vesicle. Shown are means ± SEM for three biological replicates. Statistical significance by one-way ANOVA with Dunnett’s test: *, *P* < 0.05; **, *P* < 0.01; ***, *P* < 0.001.

Given the pleiotropic effects of AFAs on S. aureus, which include increased membrane fluidity, altered proteome, and reduced surface hydrophobicity (16, 23, 24), we reasoned that these differences would be reflected in the composition of MVs released in the presence of AFAs. Accordingly, flow cytometry analysis of FM4-64-stained LAC MVs uncovered that MVs purified in the presence of LA displayed a twofold increase in lipid amounts (Fig. 5A). These MVs also had twofold-increased nucleic acid cargos (presumably RNA), as revealed by SYTO 9 staining (Fig. 5B). Taken together, these results indicate that S. aureus does not only increase MV production but also modulates MV composition in response to LA.

**FIG 6:**
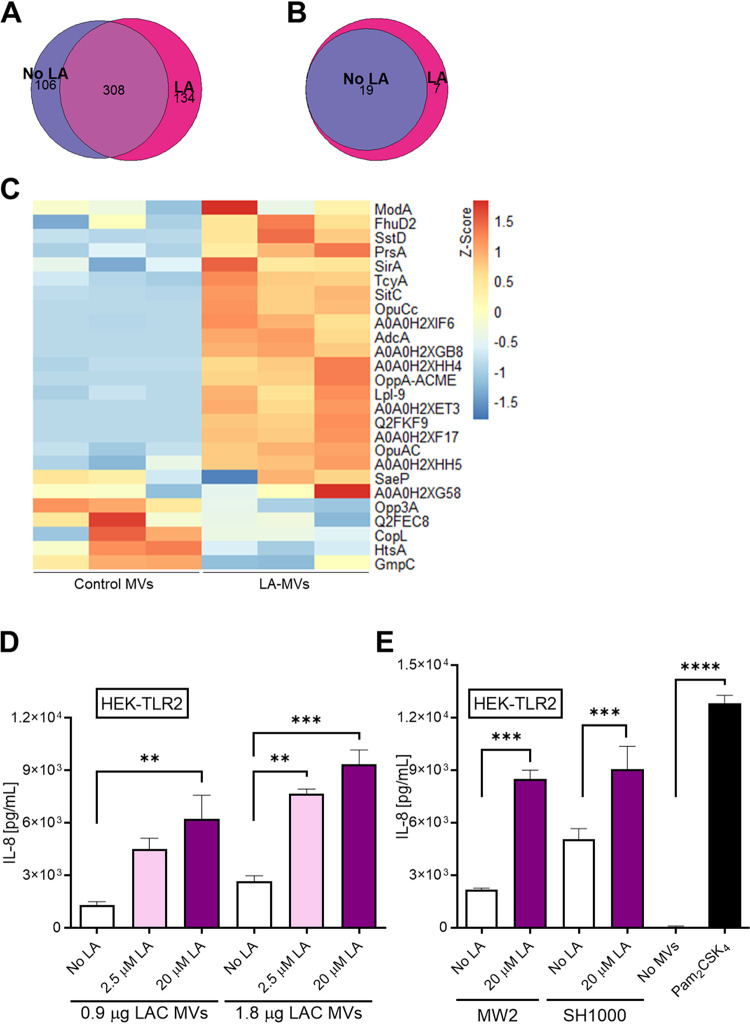
S. aureus MVs released in the presence of LA are lipoprotein enriched. (A and B) Venn diagrams displaying the numbers of proteins (A) or lipoproteins (B) detected in S. aureus LAC MVs released in the presence of 0 μM (control MVs) or 40 μM LA (LA-MVs). (C) Heatmap depicting the Z-scores of lipoproteins in control or LA-MVs. For each lipoprotein, blue indicates relatively low abundance, while red depicts high abundance. (D) Control or LA-MVs of LAC (D) and MW2 and SH1000 (E) strains were used to stimulate HEK-TLR2 cells from which supernatants were then collected and assayed for IL-8. Cells left without MVs or treated with Pam_2_CSK_4_ were used as negative and positive controls, respectively. Data shown as bar graphs are means plus SEM (*n *= 3). Statistical significance by one-way ANOVA with Tukey’s test: **, *P* < 0.01; ***, *P* < 0.001; ****, *P* < 0.0001.

We further probed the altered composition of S. aureus MVs following bacterial exposure to LA by label-free proteomics. MVs purified from S. aureus USA300 LAC grown with no LA (control MVs) or 40 μM LA (LA-MVs) significantly differed in their protein content. We detected 414 and 442 proteins in control MVs and LA-MVs, respectively, of which 308 were common to both types of MVs, and roughly one in five proteins was detected exclusively in control MVs or LA-MVs (Fig. 6A). Furthermore, compared to control MVs, more membrane proteins were identified in LA-MVs (Fig. S5A). Seven of these membrane proteins appeared to be lipoproteins, which were absent in control MVs (Fig. 6B). Quantitative analysis of protein abundance revealed that more than one in two proteins were differentially abundant (adjusted *P* value < 0.05 and |fold change| > 3) in LAC-MVs compared to control MVs (see Data Set S1 and Fig. S5B in the supplemental material). Strikingly, most of the detected lipoproteins were more abundant in LA-MVs (Fig. 6C). For the lipoprotein SitC (also referred to as MntC), the proteomic results were confirmed with control and LA-MVs isolated from a LAC strain expressing SitC with a C-terminally linked His tag. SitC-His was mildly but consistently more abundant in LA-MVs compared to control MVs (Fig. S6). These data collectively demonstrate that LA-MVs comprise an increased amount of lipoproteins.

10.1128/mSphere.00804-20.5 FIG S5 Proteomic analysis of MVs from S. aureus grown with or without LA. *S aureus* LAC MVs released in the presence of 0 μM (control) or 40 μM LA were analyzed by LC-MS/MS for their protein content. (A) Subcellular localization of the proteins detected in control or LA-MVs. (B) Volcano plot of the quantitative proteomic analysis of MV proteins from LA-treated compared to control bacteria. The relative abundance (log_2_ fold change; *x* axis) and statistical significance (−log_10_ adjusted *P* value; *y* axis) of each detected protein are depicted. Proteins with at least a threefold change in abundance and an adjusted *P* value of <0.05 are highlighted in magenta. Download FIG S5, TIF file, 1.0 MB. Copyright © 2020 Kengmo Tchoupa and Peschel. 2020 Kengmo Tchoupa and Peschel This content is distributed under the terms of the Creative Commons Attribution 4.0 International license.

10.1128/mSphere.00804-20.6 FIG S6 Increased SitC-His inclusion into MVs released in the presence of LA. MVs were purified from SitC-His-expressing bacteria grown in the presence of 0 to 10 μM LA. These MVs were stained with either a PE anti-His tag antibody or its isotype control. Shown are flow cytometry histograms representative of two independent experiments with similar results. Download FIG S6, TIF file, 0.6 MB. Copyright © 2020 Kengmo Tchoupa and Peschel. 2020 Kengmo Tchoupa and Peschel This content is distributed under the terms of the Creative Commons Attribution 4.0 International license.

10.1128/mSphere.00804-20.7 DATA SET S1 Differentially abundant proteins in MVs from S. aureus grown with or without LA. Download Data Set S1, XLSX file, 0.08 MB. Copyright © 2020 Kengmo Tchoupa and Peschel. 2020 Kengmo Tchoupa and Peschel This content is distributed under the terms of the Creative Commons Attribution 4.0 International license.

As LA-MVs were enriched in TLR2-activating lipoproteins, we investigated their capacities to stimulate TLR2-transfected HEK293 cells (HEK-TLR2). In agreement with their lipoprotein content, LA-MVs had a substantially higher capacity to stimulate HEK-TLR2 cells compared to the same amounts of control MVs, as determined by interleukin 8 (IL-8) release in response to USA300 LAC MVs (Fig. 6D) and USA400 MW2 and SH1000 MVs (Fig. 6E). Taken together, these data reveal that the TLR2-activating capacities of MVs are exacerbated, when lipoprotein-rich MVs are released in the presence of subinhibitory amounts of AFAs.

## DISCUSSION

Host-specific AFAs are important colonization barriers deployed by the innate immune system. Indeed, AFAs can inhibit growth or kill several opportunistic or pathogenic bacteria (12), including S. aureus. This opportunistic pathogen often colonizes the skin of patients with atopic dermatitis (AD), a chronic inflammatory skin disease predisposing to recurrent skin infections (7). Intriguingly, S. aureus-colonized AD patients have decreased amounts of antimicrobial sapienic and oleic acids (5, 29), suggesting that the reduced exposure of S. aureus to AFAs contributes to AD pathophysiology. The importance of AFAs at the host-pathogen interface is further demonstrated by the wide variety of resistance strategies used by S. aureus against AFAs (9, 18–21, 24). Moreover, subinhibitory amounts of AFAs induce increased resistance by mechanisms that have not been fully understood (20, 23). In the present study, we demonstrate that S. aureus responds to AFA exposure by boosting the release of MVs that protect against AFA toxicity. The MV-mediated resistance to AFAs was neither strain specific nor restricted to a limited set of AFAs. However, MVs released in response to AFAs provoked an increased TLR2-mediated immune response.

Mice immunized with S. aureus MVs are protected against otherwise lethal S. aureus lung infections in a TLR2-dependent manner (30). Beside this protective role, TLR2 activation is thought to contribute to the exacerbation and persistence of skin inflammation during AD (31, 32). In keeping with this, S. aureus MVs can cause or worsen AD-like skin inflammation in mice (33–35). Therefore, it is enticing to speculate that reduced AFA concentrations in AD skin are too low to kill S. aureus but increase the release of TLR2 agonists, which further exacerbate skin inflammation.

Extracellular resistance mechanisms to antimicrobials have been reported for Gram-negative and Gram-positive bacteria and are collectively referred to as antibiotic interceptors (36). For instance, a small protein known as lipocalin is released by Burkholderia cenocepacia to sequester hydrophobic antibiotics and enable bacterial growth in the presence of otherwise inhibitory concentrations of these drugs (37). Other protein interceptors are released as MV components and include β-lactamases from Moraxella catarrhalis and S. aureus (38, 39). Besides antimicrobial degradation, the outer MVs of Gram-negative bacteria are well characterized for their role as decoys for membrane-targeting agents like antimicrobial peptides (40–42). In contrast, not much is known about MV decoys from Gram-positive bacteria. Recently, MVs were shown to protect S. aureus against daptomycin, a membrane-targeting antibiotic (27). This bacterium releases membrane phospholipids and MVs in response to daptomycin (43). Importantly, daptomycin-induced lipid release by S. aureus is enhanced in the presence of AFAs (44), which is in agreement with our current findings that staphylococcal MV production is increased by AFAs. Notably, electron microscopic examinations of AFA-treated bacteria have consistently revealed abundant nanostructures reminiscent of MVs in Gram-positive staphylococci and streptococci (45, 46) as well as in Gram-negative Porphyromonas gingivalis and Helicobacter pylori (47, 48). Thus, MVs may represent “public goods” used by bacterial communities as a resistance mechanism against AFAs and lipophilic antibiotics such as daptomycin.

S. aureus responds to sublethal amounts of various AFAs by altering its secretome (23, 26). Interestingly, proteins secreted in response to AFAs are components of S. aureus MVs (28, 49). Our current data demonstrate that S. aureus indeed augments MV release in the presence of AFAs. The turgor pressure provides the energy for the budding of MVs (28), and this process is probably facilitated by altering membrane fluidity. In addition to AFAs, daptomycin has been reported to induce MV release in S. aureus (43). Furthermore, surfactant-like small peptides, phenol-soluble modulins (PSMs), have been shown to increase membrane fluidity of S. aureus, which favors MV budding (28). Similarly, it is likely that the fluidifying effect of AFAs on the S. aureus membrane (16, 17) would promote MV formation. Soaps and body lotions with surfactant-like properties may also promote MV release from skin bacteria and thereby increase TLR2 activation and inflammation with critical consequences in AD.

S. aureus oleate hydratase (OhyA) is another resistance strategy used by the bacterium to detoxify AFAs containing *cis*-9 double bonds (22). Intriguingly, our proteomic data revealed that OhyA was abundant in LA-MVs but absent in control MVs (see Data Set S1 in the supplemental material), suggesting that LA-MVs could detoxify AFAs not only by sequestration but also by inactivation. An additional link between MV and known anti-AFA defenses is provided by the effector and regulator of fatty acid resistance (FarE and FarR, respectively). FarE mediates AFA efflux under the control of its transcriptional regulator FarR (20). Constitutive activation of FarE confers increased resistance against AFAs and the membrane-targeting antibiotic rhodomyrtone (20, 50). Remarkably, high FarE levels also lead to an increased release of PSMs (50). Since PSMs are known to promote MV release (28, 51), we surmise an indirect contribution of FarE to this process. There is another precedent for an efflux pump-mediated lipid release in response to AFAs in Acinetobacter baumannii (11).

Besides their role as decoys for AFAs, S. aureus MVs are potent TLR2 activators by virtue of their lipoprotein cargos (28). Interestingly, mice respond to S. aureus skin infections by increasing their AFA production in a TLR2-dependent manner (52). Enhanced arachidonic acid blood levels have also been observed in mice nasally challenged with Streptococcus pneumoniae (53). *In vitro* studies have demonstrated an increased TLR2-mediated immune response to AFA-fed S. aureus (15). In light of our data, it seems likely that lipoprotein-enriched MVs released in the presence of AFAs enhanced TLR2 activation. It remains unclear why LA-MVs contain increased amounts of lipoproteins compared to control MVs. In line with our observation, S. aureus exposure to subinhibitory AFA concentrations has been found to increase the expression of several lipoproteins (23, 24). AFAs lead to upregulation of the virulence regulator SarA (23, 24), which was recently shown to control the expression of many lipoproteins (54). In keeping with this, SarA was more abundant in LA-MVs compared to control MVs. It is unclear whether certain lipoproteins have a protective role against AFAs as some of them do against copper (55) or classical antibiotics (56–58).

In sum, our data support the idea that MV release is not restricted to MRSA. As MV-conferred resistance to AFAs entails no strain specificity, it is enticing to speculate that MVs represent a conserved yet flexible strategy that S. aureus uses against structurally unrelated, hydrophobic, antimicrobial compounds. It is appropriate that our TLR2-mediated immune response appears to better recognize AFA-exposed bacteria (15) and MVs, which may help to protect healthy skin, but may exacerbate skin inflammation in AD patients. Thus, AFAs are major components of the intricate host defenses, which represent untapped resources for new antimicrobial therapeutic interventions.

## MATERIALS AND METHODS

### Bacterial strains and growth conditions.

S. aureus strains used are listed in Table S1 in the supplemental material and were routinely grown aerobically in tryptic soy broth (TSB) overnight at 37°C prior to each experiment unless stated otherwise.

10.1128/mSphere.00804-20.8 TABLE S1 Staphylococcus aureus strains used in this study. Download Table S1, PDF file, 0.06 MB. Copyright © 2020 Kengmo Tchoupa and Peschel. 2020 Kengmo Tchoupa and Peschel This content is distributed under the terms of the Creative Commons Attribution 4.0 International license.

### Membrane vesicle purification.

MVs were isolated with the ExoQuickTC kit (EQPL10TC; System Bioscience) as described elsewhere (28). Briefly, overnight bacterial cultures diluted to an optical density at 600 nm (OD_600_) of 0.1 in 20 ml plain TSB or TSB supplemented with AFAs or their solvent dimethyl sulfoxide (DMSO) were grown with shaking for 6 h (late exponential growth phase). Next, bacteria were pelleted by centrifugation, and supernatants were sterile filtered. MVs in these culture filtrates were concentrated with 100-kDa centrifugal concentrator cartridges (Vivaspin 20; Sartorius) prior to precipitation with the ExoQuickTC kit and resuspension in 500 μl phosphate-buffered saline (PBS).

### Protein, lipid, and nucleic acid quantification in MVs.

The quantification of the protein fraction in purified MVs was performed using a Bradford assay following the manufacturer’s recommendations (Quick Start Bradford protein assay kit; Bio-Rad). For lipids, the lipophilic dye FM4-64 (Life Technologies) was used to stain MVs for 10 min at a final concentration of 5 μg/ml. The nucleic acid cargo of MVs was assessed via staining with 10 μM SYTO 9 for 30 min. Samples were analyzed with a CLARIOStar microplate reader (BMG Labtech) or a BD LSRFortessa flow cytometer (BD).

### SitC detection.

SitC expression in S. aureus USA300 LAC Δ*spa* pTX SitC-His was induced with 0.5% xylose added to TSB without glucose supplemented with DMSO or linoleic acid. After bacterial growth, MVs were purified and their protein content was quantified as described above. Thirty micrograms (protein amount) per MV sample were stained with either a phycoerythrin (PE) anti-His tag antibody (clone J095G46; BioLegend) or its appropriate mouse IgG2a, κ PE isotype control antibody (clone MOPC-173; BioLegend). PE-labeled MVs were then analyzed by flow cytometry.

### Click chemistry with linoleic acid alkyne.

Exponentially growing bacteria were centrifuged and resuspended in either sterile, fresh TSB or S. aureus-conditioned medium. These bacteria or purified MVs were incubated at 37°C for 5 to 20 min with 20 μM linoleic acid alkyne (Cayman Chemical). Samples were then centrifuged, and pellets were resuspended in Click-iT cell reaction buffer supplemented with copper(II) sulfate and Click-iT cell buffer additive, as recommended by the manufacturer (Click-iT cell reaction buffer kit; Invitrogen). Click chemistry was performed at 25°C for 30 min with 7 μM azide fluor 488 (Merck). After washing with PBS, LA-stained bacteria or MVs were analyzed by flow cytometry.

### Growth curves.

Overnight bacterial cultures were diluted to an OD_600_ of 0.05 in plain MHB or MHB supplemented with AFAs and/or 3.75 to 60 μg/ml MVs. Bacteria were then grown in a 96-well plate (U-bottom) at 37°C with linear shaking at 567 cpm (3-mm excursion) for 20 h, and the OD_600_ was measured every 15 min with an Epoch 2 plate reader (BioTek). Areas under the curves were computed with GraphPad Prism 8.4.2.

### Growth inhibition assays.

After dilution to an OD_600_ of 0.001 in MHB and treatment with DMSO (no LA), LA, or LA plus 30 μg/ml MVs, bacteria were either directly plated or grown for 2 h at 37°C before plating on tryptic soy agar and CFU counting.

### Quantitative label-free proteomics.

For proteomic analysis, MVs were isolated as described above from S. aureus USA300 LAC strain grown in TSB supplemented with DMSO (control MVs) or 40 μM LA (LA-MVs). After protein quantification with a Bradford assay, 20 μg per biological replicate was run on a gel until all the proteins had moved from the stacking gel to the resolving gel. After tryptic in-gel digestion, samples were analyzed by liquid chromatography-tandem mass spectrometry (LC-MS/MS) as described elsewhere (28, 59). Briefly, a Q Exactive HF Hybrid Quadrupole-Orbitrap mass spectrometer (Thermo Scientific) and a 90-min LC separation with an EASY-nLC 1200 system (Thermo Scientific) were employed. The data were used to interrogate the UniProt Staphylococcus aureus USA300 database UP000001939, and the common contaminant database from MaxQuant (60). Protein identification and quantification were performed with the MaxQuant software using default settings. Intensities were log_2_ transformed with the Perseus software, and proteins with only one or no valid value for every sample in triplicate were filtered. Missing values were then put in as the lowest intensity across all samples in R. Differential protein abundance was calculated with the limma R package (61).

### HEK-TLR2 cell culture and stimulation.

HEK293 cells stably transfected with the human TLR2 gene (HEK-TLR2) (Invivogen) were maintained in Dulbecco’s modified Eagle’s medium (DMEM) supplemented with 10% fetal calf serum (FCS), 100 μg/ml Normocin, and 10 μg/ml blasticidin. For stimulation experiments, HEK-TLR2 cells were seeded into 24-well plates (2 × 10^5^ cells/well), and cultivated until confluence was reached (2 to 3 days). Next, the growth medium was removed, cells were washed once with PBS before incubation for 20 h with MVs diluted in 500 μl DMEM per well. Human IL-8 release was used as a proxy for TLR2 activation and measured using an enzyme-linked immunosorbent assay (ELISA) kit (R&D Systems) according to the manufacturer’s instructions. The synthetic lipopeptide Pam_2_CSK_4_ (200 ng/ml) was used as a positive control.

### Statistical analysis.

Except for the proteomics data, statistical tests specified in the figure legends were performed with GraphPad Prism 8.4.2, and *P* values of < 0.05 were considered significant. Analysis of variance (ANOVA) with Dunnett’s or Tukey’s multiple-comparison test was used. The fold changes and *P* values of the proteomics data were calculated with the R package limma (61), with control MVs as the reference.

### Data availability.

The mass spectrometry proteomics data have been deposited to the ProteomeXchange Consortium via the PRIDE (62) partner repository with the data set identifier PXD018809, where control MVs are labeled R01, R02, and R03. All other data generated are available within the paper and the supplemental material files.
