# Supplementary figures and images for: Staphylococcus aureus Releases Proinflammatory Membrane Vesicles To Resist Antimicrobial Fatty Acids
Source: mSphere. 2020 Sep 30;5(5):e00804-20. doi: 10.1128/mSphere.00804-20 (PMC7529438; doi:10.1128/mSphere.00804-20)

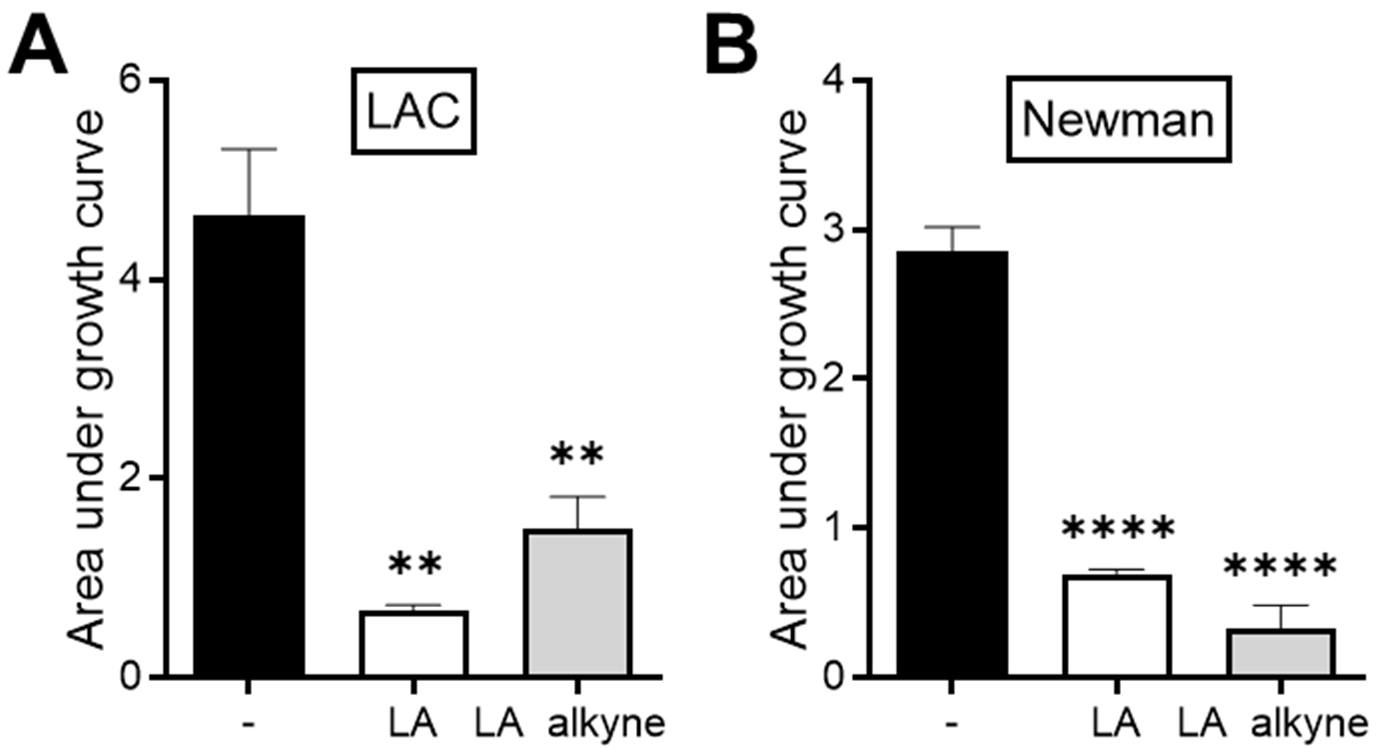

Supplement: FIG S1 [file mSphere.00804-20-sf001.tif]

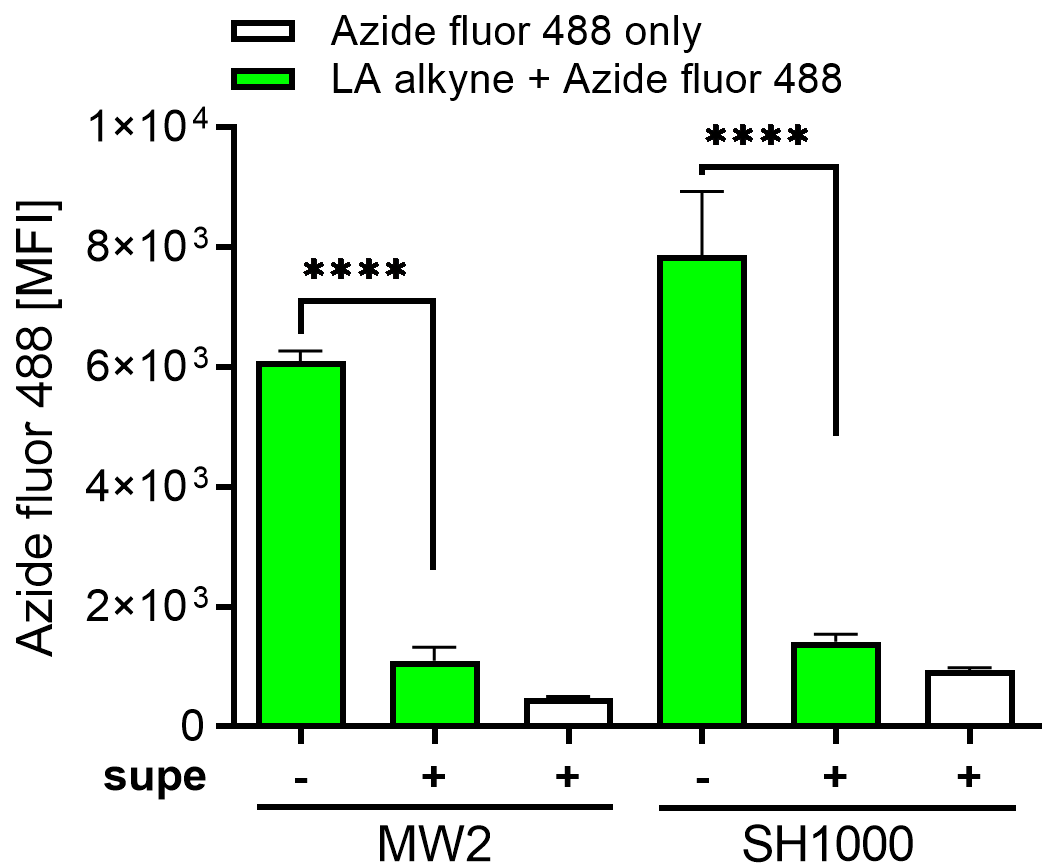

Supplement: FIG S2 [file mSphere.00804-20-sf002.tif]

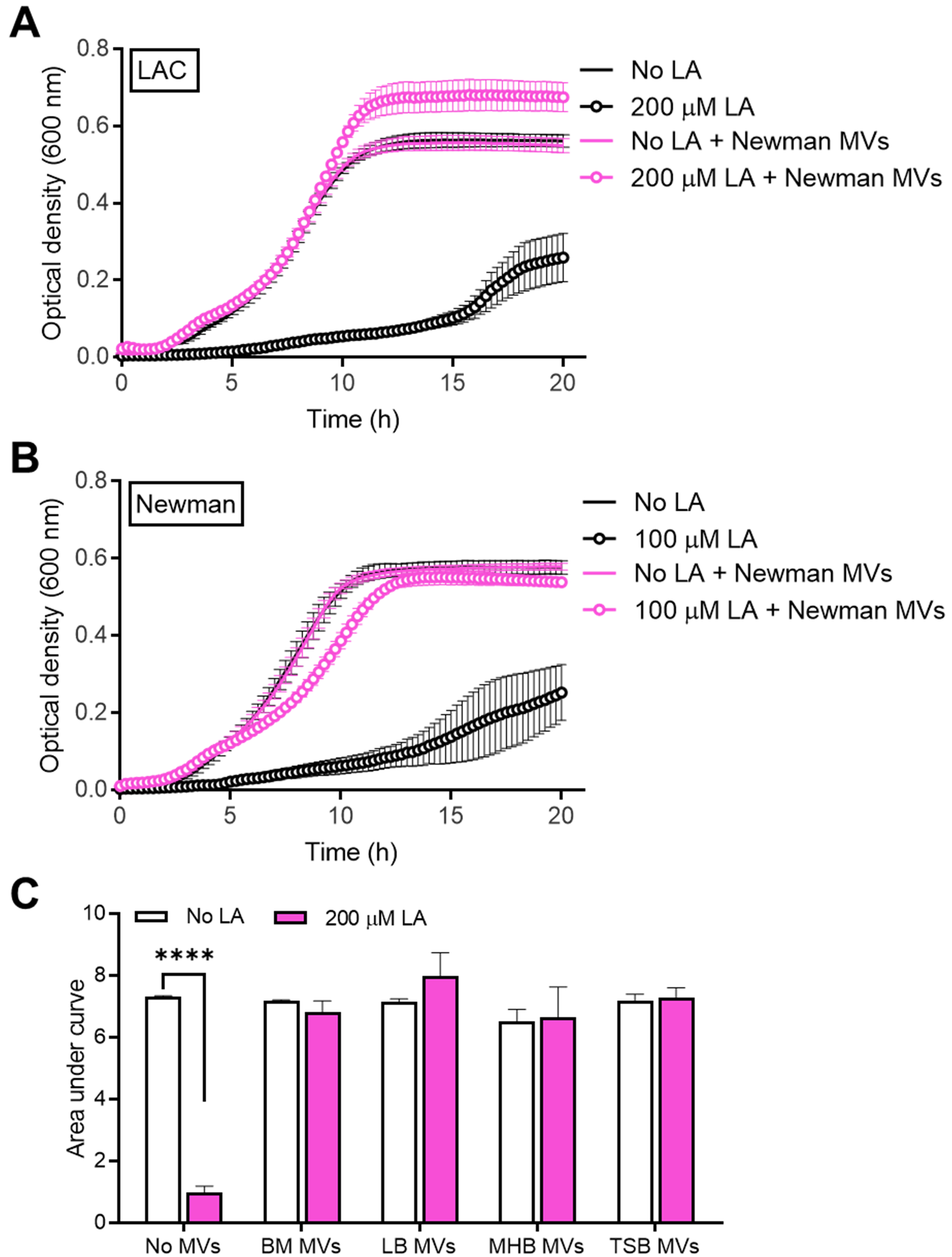

Supplement: FIG S3 [file mSphere.00804-20-sf003.tif]

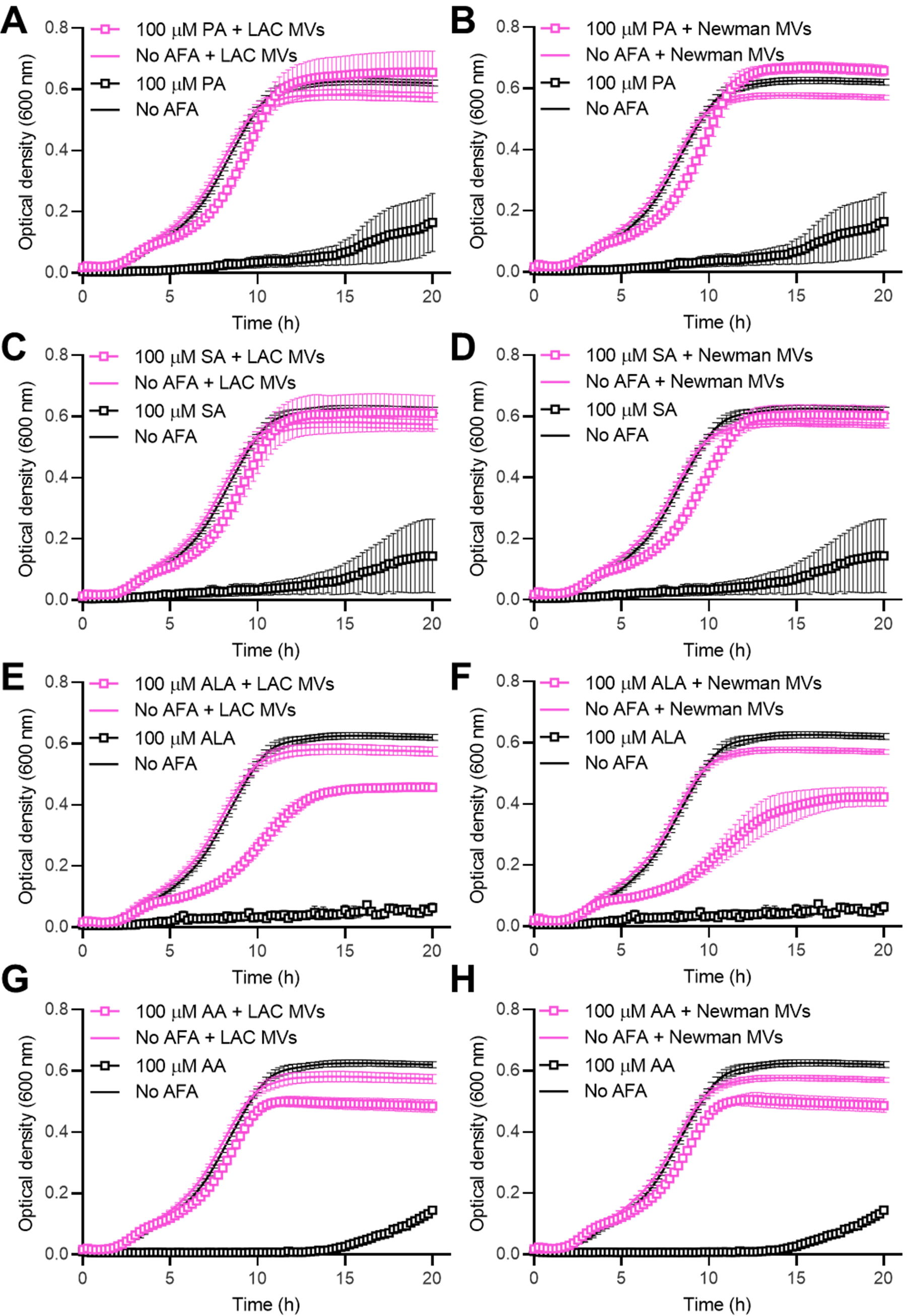

Supplement: FIG S4 [file mSphere.00804-20-sf004.tif]

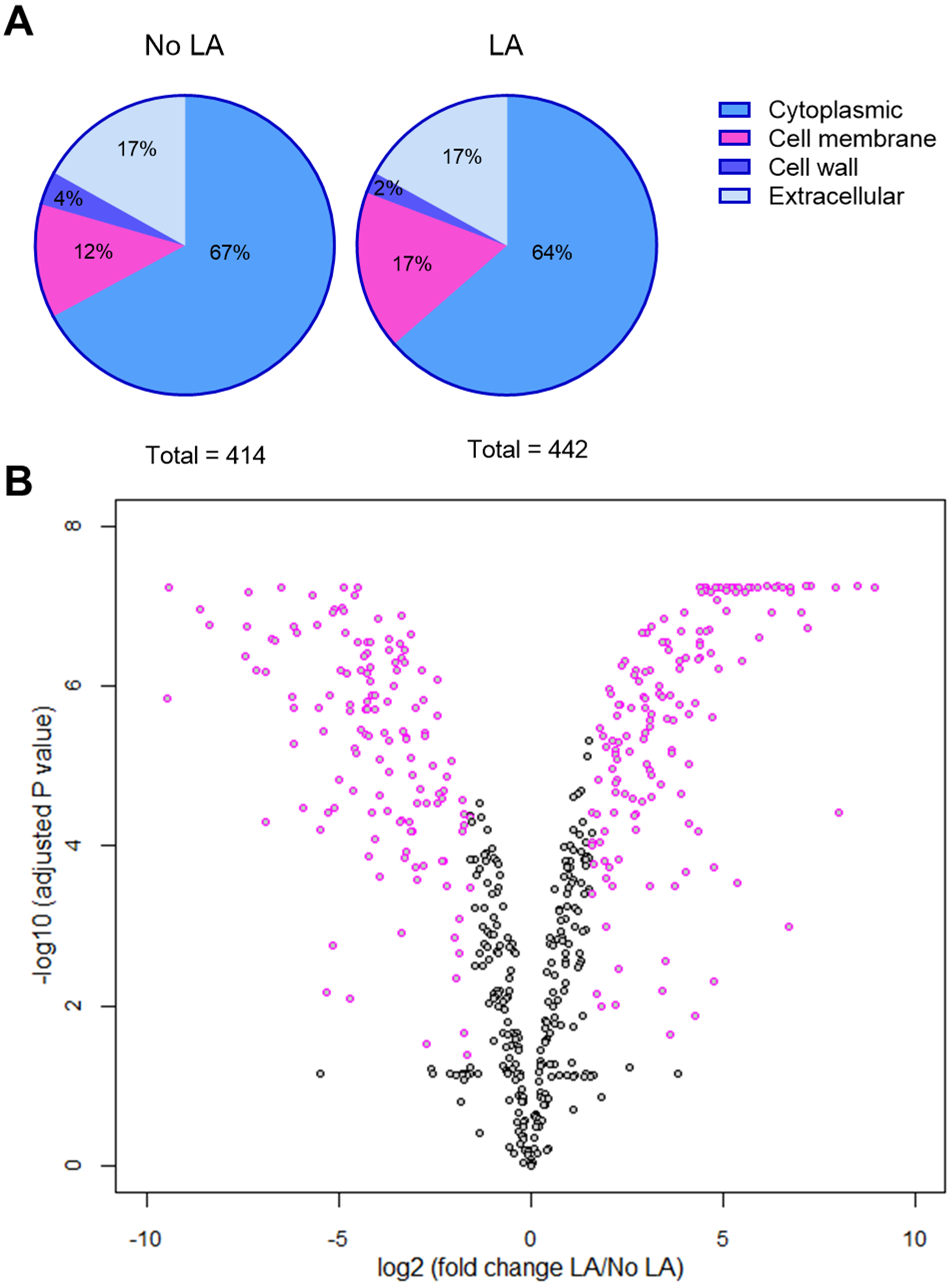

Supplement: FIG S5 [file mSphere.00804-20-sf005.tif]

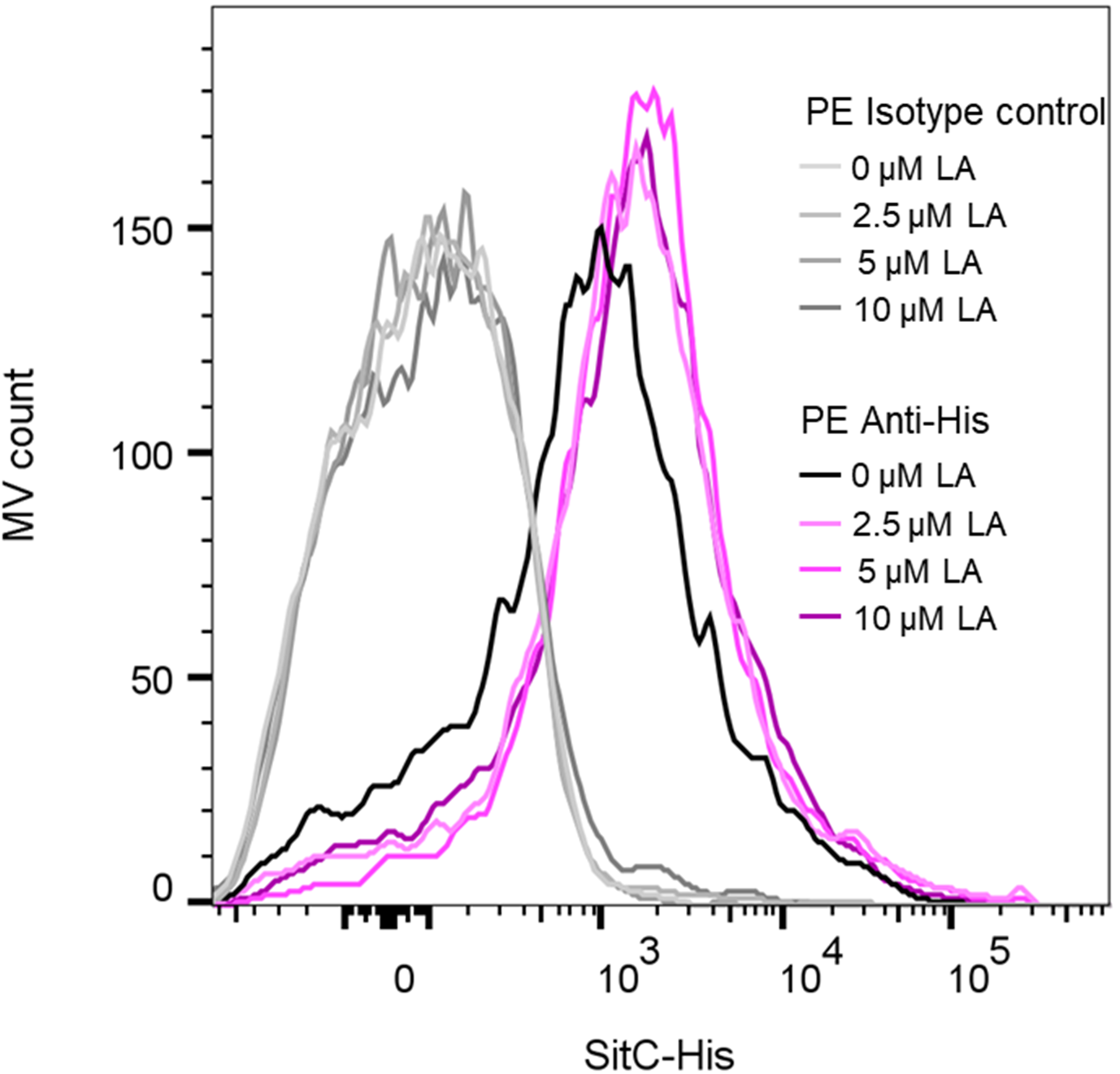

Supplement: FIG S6 [file mSphere.00804-20-sf006.tif]
