# Supplementary material for: Staphylococcus aureus Releases Proinflammatory Membrane Vesicles To Resist Antimicrobial Fatty Acids
Source: mSphere. 2020 Sep 30;5(5):e00804-20. doi: 10.1128/mSphere.00804-20 (PMC7529438; doi:10.1128/mSphere.00804-20)
Supplement: TABLE S1 [file mSphere.00804-20-st001.pdf]

Table S1. *Staphylococcus aureus* strains used in this study

Strain	Description	Source or reference
USA400 MW2	Community-acquired MRSA (CA-MRSA)	Michael Otto (Wang et al., Nat Med 13:1510–1514, 2007, https://doi.org/10.1038/nm1656)
USA300 LAC	CA-MRSA	
LAC Δ*agr*	*S. aureus* USA300 LAC defective for Agr	
LAC Δ*spa* pTX-SitC-His	*S. aureus* USA300 LAC defective for the protein A and expressing His-tagged SitC	Schlatterer et al., mBio 9:e01851-18, 2018, https://doi.org/10.1128/mBio.01851-18
Newman	Methicillin-sensitive *S. aureus* (MSSA)	Duthie and Lorenz, J Gen Microbiol 6: 95–107, 1952
SH1000	NCTC8325 derivative with a functional *rsbU* gene, Δ*tcaR*, cured of φ11, φ12, and φ13; MSSA	Simon Foster (Horsburgh et al., J Bacteriol 184: 5457–5467, 2002, DOI: 10.1128/jb.184.19.5457-5467.2002)
